# 8-Hydroxy-2’-deoxyguanosine protein immunoexpression is associated with the pathogenesis of actinic cheilitis

**DOI:** 10.1016/j.abd.2023.06.010

**Published:** 2024-02-24

**Authors:** Cíntia Barreto de Oliveira Varela, Cristianne Kalinne Santos Medeiros, Jabes Gennedyr da Cruz Lima, Éricka Janine Dantas da Silveira, Patrícia Teixeira de Oliveira

**Affiliations:** Postgraduate Program in Dental Sciences, Department of Dentistry, Universidade Federal do Rio Grande do Norte, Natal, RN, Brazil

*Dear Editor,*

Actinic Cheilitis (AC) is a potentially malignant, chronic inflammatory condition that can progress to lip squamous cell carcinoma. Ultraviolet radiation is the main factor associated with the development of AC.^1^, ^2^, ^3^ Chronic exposure to ultraviolet radiation leads to the generation of reactive oxygen species that can cause oxidative modifications in DNA bases, mainly in guanine, generating 8-Hydroxy-2'-Deoxyguanosine (8-OHdG) which can trigger gene mutations. Therefore it has been used as a sensitive marker of oxidative damage and as a prognostic factor for skin cancer, lung cancer, esophageal cancer, breast cancer, colon cancer and lymphoma, among others.^4^, ^5^

The present research consisted of a retrospective, descriptive and semi-quantitative study of the immunohistochemical expression of the 8-OHdG protein in 57 cases of AC specimens. The study was conducted at the Department of Dentistry, Universidade Federal do Rio Grande do Norte, Natal, RN, Brazil, and was approved by the Ethics Committee of the Federal University of Rio Grande do Norte (Protocol nº 3.476.834).

For the morphological study, 5-µm sections were cut from the paraffin-embedded material, mounted on glass slides, and stained with hematoxylin-eosin. The histological sections were examined and classified as without or with epithelial dysplasia and graded as mild, moderate, or severe according to the World Health Organization’s (WHO) morphological criteria.^6^

For immunohistochemical analysis, the material treated with the anti-8-OHdG antibody (Santa Cruz Biotechnology Cat# sc-66036, RRID: AB 832272) was examined by two previously calibrated and blinded evaluators. Analysis was performed by light microscopy at 100× and 400× magnification in the epithelial component throughout the lesion whose extent was determined by the area of solar elastosis in connective tissue and by epithelial alterations if present. The cases were categorized based on an immunoreactivity score. For this purpose, the intensity of staining was first evaluated in the epithelium of the lesion and classified as follows: 0 (negative), 1 (weak), 2 (moderate), and 3 (strong). The predominant intensity in each case was considered. Immunostaining was analyzed semi-quantitatively by assigning the following scores: 0 (negative), 1 (1% to 50% positive cells), and 2 (51% to 100% positive cells). Epithelial cells exhibiting brown staining in the nucleus and/or cytoplasm were classified as positive.^5^, ^7^ The immunoreactivity score was then calculated by multiplying the score for staining intensity by the score for the percentage of positive cells. The final score of each case ranged from 0 to 6, where 0‒2 = weak immunoreactivity, 3‒4 = moderate immunoreactivity, and 5‒6 = strong immunoreactivity.

There was a predominance of male (72.2%) and white patients (64.9%), non-smokers or former smokers (49.2%), aged 40 years or older (93%) and with a history of chronic exposure to ultraviolet radiation (66.7%). White spots were the most frequent clinical feature in the cases studied (56.1%), followed by white plaque and desquamation; ulceration was less common (10.5%) (Table 1).

**Table 1:** Distribution of the sample according to demographic data and clinical characteristics of actinic cheilitis.

	n	%
**Sex**		
Male	44	72.2
Female	13	22.8
**Age (years)**		
<40	4	7.0
≥40	53	93.0
**Race**^a^		
White	37	64.9
Black	15	26.3
**Smoking**^a^		
Non-smoker	11	19.3
Smoker	4	7.0
Former smoker	17	29.8
**Desquamation**		
Yes	27	47.4
No	30	52.6
**White spot**		
Yes	32	56.1
No	25	43.9
**White plaque**		
Yes	28	49.1
No	29	50.9
**Erythema**		
Yes	25	43.9
No	32	56.1
**Ulcer**		
Yes	6	10.5
No	51	89.5

a Lost data: Race ‒ 5 (8.8%) cases; Smoking ‒ 25 (43.9%) cases.

Immunohistochemical positivity in epithelial cells was observed in 100% of the cases analyzed, especially in the basal and parabasal layers of the epithelium. Immunostaining was predominantly nuclear with mild cytoplasmic staining in 39.6% of cases and predominantly cytoplasmic staining in 12.1%, while the same intensity was observed in the nucleus and cytoplasm of 48.3%. Strong immunoreactivity to the antibody was detected in 64.9% of the cases (Fig. 1). The observation of immunostaining in the nucleus and cytoplasm may be explained by the fact that 8-OHdG is found in both nuclear and mitochondrial DNA.^8^, ^9^

**Figure 1:**
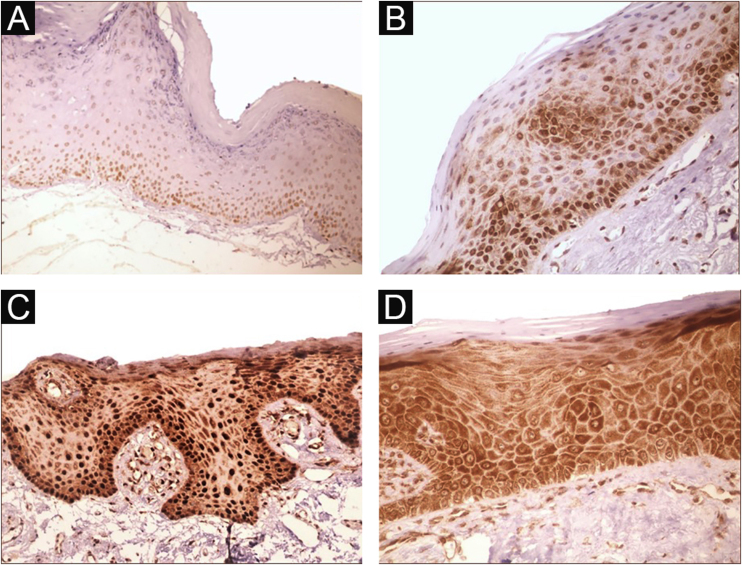
Expression profile of 8-OHdG in Actinic Cheilitis (AC). (A) Immunoreactivity score 1 in AC without epithelial dysplasia (100×). (B) Immunoreactivity score 2 in AC with mild epithelial dysplasia. Note the predominance of nuclear immunostaining (200×). (C) Immunoreactivity score 3 in AC with moderate epithelial dysplasia. Note the predominance of nuclear immunostaining (200×). (D) Immunoreactivity score 3 in AC with moderate epithelial dysplasia. Note the predominance of cytoplasmic staining (200×).

Oxidative stress on DNA was present regardless of the clinical features of the lesions. No statistically significant differences in 8-OHdG expression were found according to the clinical characteristics of the lesions (p > 0.05) (Table 2) or the presence of smoking (p = 0.266) (Table 3). Prabhulkar and Li monitored the kinetics of 8-OHdG release on the surface of a lung epithelial cell exposed to nicotine in order to evaluate the oxidative damage caused by tobacco, in which the authors observed that the higher the level of cell exposure to nicotine, the greater the oxidative damage to DNA, promoting an increase in the level of 8-OHdG secreted by the cell.^8^ In our samples, oxidative DNA damage demonstrated by the presence of 8-OHdG was observed irrespective of exposure of the subjects to tobacco.

**Table 2:** Sample size, median, 25^th^ and 75^th^ quartiles, and statistical significance (p) for 8-OHdG immunoreactivity score according to the clinical characteristics of actinic cheilitis.

Variable	8-OHdG immunoreactivity			
	n	Median	Q25-Q75	p
**Desquamation**				0.741
Yes	27	3.00	2.00‒3.00	
No	30	3.00	1.75‒3.00	
**White spot**				0.506
Yes	32	3.00	2.00‒3.00	
No	25	3.00	1.50‒3.00	
**White plaque**				0.479
Yes	28	3.00	1.25‒3.00	
No	29	3.00	2.00‒3.00	
**Erythema**				0.087
Yes	25	3.00	1.00‒3.00	
No	32	3.00	2.25‒3.00	
**Ulcer**				0.065
Yes	6	1.50	1.00‒3.00	
No	51	3.00	2.00‒3.00	

Mann-Whitney test.

**Table 3:** Sample size, median, 25^th^ and 75^th^ quartiles, and statistical significance (p) for final 8-OHdG immunoreactivity score according to smoking.

Variable	8-OHdG immunoreactivity			
	n	Median	Q25‒Q75	p
Smoker	4	3.00	3.00‒3.00	
Non-smoker	11	3.00	1.00‒3.00	0.266
Former smoker	17	3.00	1.50‒3.00	

Kruskal-Wallis test.

Histopathological grading revealed some degree of epithelial dysplasia in 87.7% of cases. Mild epithelial dysplasia was the most common (38.6%), followed by moderate (29.8%) and severe epithelial dysplasia (19.3%). Epithelial dysplasia was absent in 12.3% of cases. Strong immunoreactivity was observed in most cases, regardless of the presence of epithelial dysplasia or the grade of severity. No significant difference in 8-OHdG expression was observed between the different degrees of oral epithelial dysplasia (p = 0.225) (Table 4). Similarly, Yoshifuku et al. also found a predominance of strong 8OHdG immunoreactivity in actinic keratosis lesions (presence of mild or moderate epithelial dysplasia) and Bowen’s disease (severe epithelial dysplasia).^5^ These findings might be explained by the fact that chronic exposure to ultraviolet radiation triggers the continuous production of reactive oxygen species and oxidative stress and the consequently constant formation of 8-OHdG.

**Table 4:** Sample size, median, 25^th^ and 75^th^ quartiles, and statistical significance (p) for 8-OHdG immunoreactivity score according to WHO grading of oral epithelial dysplasia.

WHO grading	8-OHdG immunoreactivity			
	n	Median	Q25‒Q75	p
Without dysplasia	7	3.00	3.00‒3.00	
Mild epithelial dysplasia	22	3.00	2.00‒3.00	0.225
Moderate epithelial dysplasia	17	3.00	3.00‒3.00	
Severe epithelial dysplasia	11	3.00	2.50‒3.00	

Kruskal-Wallis test.

Although 8-OHdG expression is useful for the evaluation of oxidative DNA damage and is well established as a prognostic indicator in different types of cancer, with evidence suggesting that oxidative DNA damage through the formation of 8-OHdG is involved in the pathogenesis and progression of squamous cell carcinoma,^7^, ^8^, ^10^ the present results point to difficulties in using this marker to establish an association with clinicodemographic characteristics and with the morphological severity of actinic cheilitis since 8-OHdG was markedly expressed in most of the cases analyzed.

The results of this study confirm that oxidative stress through DNA damage plays a role in AC as suggested by the strong immunohistochemical expression of 8-OHdG in the cases analyzed. However, the lack of a significant difference in anti-8-OHdG immunoreactivity between different degrees of oral epithelial dysplasia and clinicodemographic factors demonstrates that this marker is altered regardless of clinical characteristics and degree of epithelial dysplasia and the oxidative DNA base modifications is an early event in lip carcinogenesis. Thus, adjuvant drug treatments that minimize the damage caused by oxidative stress, such as topical antioxidant agents, may be useful therapeutic measures in patients with AC.

## Financial support

None declared.

## Authors’ contributions

Cíntia Barreto de Oliveira Varela: Data collection, analysis and interpretation; Critical literature review; Preparation and writing of the manuscript; Approve the final version of the manuscript.

Cristianne Kalinne Santos Medeiros: Data collection, analysis and interpretation; Statistical analysis; Approve the final version of the manuscript.

Jabes Gennedyr da Cruz Lima: Data collection, analysis and interpretation; Approve the final version of the manuscript.

Éricka Janine Dantas da Silveira: Study conception and planning; Effective participation in research orientation; Approve the final version of the manuscript.

Patrícia Teixeira de Oliveira: Study conception and planning; Effective participation in research orientation; Approve the final version of the manuscript.

## Conflicts of interest

None declared.
